# Multi-leveled objects: color as a case study

**DOI:** 10.3389/fpsyg.2014.00592

**Published:** 2014-07-02

**Authors:** Liliana Albertazzi, Roberto Poli

**Affiliations:** ^1^Center for Mind/Brain Sciences and Department of Humanities, University of TrentoTrento, Italy; ^2^Department of Sociology and Social Research, University of TrentoTrento, Italy

**Keywords:** color appearances, color models, color systems, levels of reality, phenomenology

## Abstract

The paper presents color as a case study for the analysis of phenomena that pertain to several levels of reality and are typically framed by different sciences and disciplines. Color, in fact, is studied by physics, biology, phenomenology, and esthetics, among others. Our thesis is that color is a different entity for each level of reality, and that for this reason color generates different observables in the epistemologies of the different sciences. By analyzing color as a paradigmatic case of an entity naturally spreading over different levels of reality, the paper raises the question as to whether making explicit the usually implicit ontological assumptions embedded within the different observables exploited by the different sciences may eventually clarify some of the difficulties of developing a comprehensive theory of color.

## INTRODUCTION

What is color? Is it a quality of the phenomenal (subjective) appearance or a property of the physical object? Or both? How are the phenomenal quality and the physical property related to each other? As well known, widely different answers have been provided. Among them are the following: color realism based on the reflectance of light by surfaces, that is, physics; phenomenal objectivism based on sensory-motor contingencies; color subjectivism based on qualia, i.e., atomic color experiences; color as brain product based on the effects of objects within us, from the point of view of the nervous system; and color dispositionalism based on the effects of objects within us, from the point of view of our experiential states (on the different epistemological stances see Byrne and Hilbert, 1997).

Consequently, theories on these various matters have analyzed aspects concerning the nature of stimuli, the workings of the neurons, sensory response, the eminently qualitative nature of phenomenal vision, or aesthetic yield. But on establishing correlations among events at these various levels, one should be careful to avoid epistemologically collapsing their relations of ontological dependence into reductions to either the physical or the neuronal level (Albertazzi, 2006, 2013). In fact, as Köhler wrote

If someone states that things seen must first be experienced as if they were in the brain, he has not realized that the first part of his statement refers to the visual field as a fact of experience, whilst in the second part, where he uses the expression “the brain,” he is speaking of a physical object in physical space. This means that he expects to see parts of visual space localized in relation to parts of physical space, and this notion is entirely impossible.

(Köhler, 1947, p. 213)

The discussion on color continues to suffer from the same shortcomings as denounced by Köhler. It still lacks, for example:

• A categorical classification of the differences among the physical, the neuronal, and the properly psychic (mental) marking the onset of color perceptions.
• A distinction between the color stimuli and subjective color conditions of perceptibility (for example, the assimilative phenomena in color appearances, the role of subjective integrations, the capacity to understand such aspects of colors as the difference between warm and cold or light and heavy colors).
• A precise terminology according to the different levels of analysis, relatively to the different color “observables.”
• An explicit correlation between models of color and the specific color observables to which they refer.

The thesis put forward in this study is that *only the framework provided by a properly developed theory of levels of reality can handle the complexity of color perception and color spaces*. The assumption, however, is that the different color observables are not totally independent from one another, in the sense that they are connected by a network of dependencies arising from the different levels of reality.

As a step toward understanding and clarifying the nature of color, this paper suggests verifying whether at least some of the controversial aspects of color understanding depend on different ontological (not epistemological) assumptions. Otherwise stated, we propose to bracket the models’ epistemological assumptions as far as is possible in order to better grasp the possible presence of underlying ontological differences.

Color perception is characterized by the presence of different theories based on conflicting primitives (wavelength, neural correlates, color appearances), and parameters (hue, saturation, chroma, brightness, lightness, to mention but a few). Furthermore, a variety of color solids have been proposed as models of the space of colors, including cylindrical, conic, pyramidal, and spherical ones (Billmeyer, 1987). Moreover, even when the different theories adopt the same categories, they define them in different and often conflicting ways.

To make matters worse, even the identification of colors raises major problems: to wit, the color matching procedure, on which most colorimetry is based (Boynton, 1979; Brainard, 1995; Koenderink and van Doorn, 2003; Koenderink, 2010), exploits a severely restricted use of color terms and does not consider what the viewer actually perceives, with the exception of the viewpoint of color differences. The phenomenological aspects of observed colors (Stumpf, 1917; Hering, 1920, 1964; Gelb, 1929; Katz, 1935) remain hidden behind the yes/no responses to just noticeable differences (*jnd* – the units of psychophysical analysis).

The question also arises as how to relate natural language color terms for perceived dimensions of color, i.e., relatively to what kinds of concepts are encoded or not encoded by languages, what are the ontological referents, in what universal and linguistic (or culture-specific) meanings consist, etc. The so-called nature/nurture debate in the field of colors is particularly difficult to address, given the tangled development of the taxonomy of colors over time and in the different languages (Williams, 1976; Dedrick, 1998; Paramei, 2007; Jameson and Komarova, 2009; Rakhilina and Paramei, 2011), the terminology adopted by scientific theories that may define colors according to metrical parameters or differently shaped color spaces, and the question of how the subjective perception of color relates to cross-cultural color naming (Jameson, 2005). Furthermore, many more colors exist perceptively than can be linguistically named (Kuehni, 2007, 2010). The problem is that there are not enough terms to qualify color appearances in simple, precise, and exhaustive terms, if necessary.

Scientific nomenclatures usually adopt severely constrained sets of basic terms and qualifiers. While this may be appropriate for specific uses, such as industrial ones, it is too coarse to capture distinctions that people spontaneously make.

Color nomenclatures usually apply to isolated, uniform patches, or the very simplest configurations of color mondrians. Moreover, special color nomenclatures refer to colors pertaining to particular areas of the entire space of colors. A color nomenclature typically relies on a highly simplified framework based on a small number of qualifiers and their combinations (for instance, “red,” “deep red,” “dark red,” “light red,” etc.). These labels are the linguistic translations of numerical expressions. That is, they are operational definitions that do not consider the correlation between perception of color and the linguistic expression that best matches the perception. Perception, depending on different settings, including the physical and the mental, often leads to color terms that do not fit into acknowledged standards.

## COLORS AND COLOR TERMS

The CIE (Commission Internationale de l’Eclairage, International Commission of Illumination) definition of color runs as follows: Color (perceived) is the “characteristic of visual perception that can be described by attributes of hue, brightness (or lightness) and colourfulness (or saturation or chroma; see International Lighting Vocabulary [ILV], 2011; Standard CIE S 017/E:2011). A series of notes (http://cie.co.at/index.php?i_ca_id=827) clarify that: “when necessary, to avoid confusion between other meanings of the word, the term “perceived color” may be used (note 1); that “perceived color depends on the spectral distribution of the color stimulus, on the size, shape, structure, and surround of the stimulus area, on the state of adaptation of the observer’s visual system, and on the observer’s experience of the prevailing and similar situations of observation” (note 2); and that “perceived color may appear in several modes of color appearance. The names for various modes of appearance are intended to distinguish among qualitative and geometric differences of color perceptions. Some of the more important terms of the modes of color appearance are given in ‘object color,’ ‘surface color,’ and ‘aperture color.’ Other modes of color appearance include film color, volume color, illuminant color, body color, and Ganzfeld color. Each of these modes of color appearance may be further qualified by adjectives to describe combinations of color or their spatial and temporal relationships. Other terms that relate to qualitative differences among colors perceived in various modes of color appearance are given in ‘luminous color’, ‘non-luminous color,’ ‘related colour,’ and ‘unrelated color”’ (note 4).

However, color *terms* can only be linguistic labels of perceived *appearances* of colors, not of physical stimuli because we do not perceive physical stimuli as such. If anything, we perceive colors as a consequence of physical stimulation. Also in this respect, however, the relation between physical stimuli and color appearances is less direct than one might think, or can be taken for granted, given the strong contextual dependence of color appearances (Chevreul, 1839; Albers, 1963). It is our suggestion that grounding color nomenclature on the perceptual experience of subjects provides models more robust than those based on an automatic translation of numerical expressions or geometrical positions in a color space. From this emerges the need to arrive at a robust perceptual definition of color terms.

Natural languages use different types of color terms (Biggam, 2012). Since Berlin and Kay’s (1977) seminal book, the literature has drawn on a variety of different methodologies ranging from purely linguistic analyses (Wierzbicka, 2006), to anthropological field researches (MacLaury et al., 2007), mainly with the subministration of Munsell chips^1^ (Berlin and Kay, 1977; MacLaury, 1992; Davidoff et al., 1999), and Osgood’s semantic differential (Madden et al., 2000). More recently, results from the neurosciences have begun to be used (Kay and McDaniel, 1978; Wuerger et al., 2005). For an extensive review of the different universalist and relativist positions see Da Pos and Albertazzi (2007).

Specifically, as regards basic color terms^2^, natural languages segment color appearances according to identifiable patterns. Most languages broadly agree on the prototypicality of linguistic categories for so-called focal colors (Rosch, 1973; Rosch et al., 1976). However, agreement on what aspects are the proper referents of color terms in natural languages is still lacking, because different models refer to different parameters or different aspects of color. Most of the dispute between universalists and relativists on color terms, for example, arises because the exponents of each perspective use concepts of color referring to *different* realities, including stimuli, neural correlates, and color appearances. The usual recourse in these cases to qualifiers such as “‘unique,” “pure,” “primary,” “elementary,” “basic,” “focal,” and “prototypical” is widely insufficient, because these qualifiers are themselves far from being univocal. A more systematic framework is needed.

To present one of the customary confusions in addressing colors, it is enlightening to consider the difference between hue and color. Unique (also known as unitary or psychologically primary) colors (Hering, 1920) are colors which do not resemble any other colors, whilst binary, or psychologically mixed colors resemble at least two others. The definition is based on the visual similarity which a color shows, or does not show, with other colors, obtained by pure phenomenological observation. The system of color notation closest to the perception of colors based on their visual similarity is the Natural Color System (NCS, Sivik, 1991). In the NCS, reference to unique hues amounts to reference to yellow, red, blue, and green, while reference to unique colors includes also the achromatic white and black; in fact, from a phenomenological viewpoint, black and white are also perceived as colors. The categories of color and hue are not easily definable, however. Prima facie we might define color as everything that is directly seen, i.e., as the color appearance – defined in CIE as the “aspect of visual perception by which things are recognized by their color” – while hue is the aspect possessed by many colors and which makes them chromatic, distinguishing them from non-chromatic colors. A specific hue is more or less visible in a particular color, in the sense that two colors can be of the same hue: one can see the presence of more red in a highly chromatic color of red hue than in a scantily chromatic color of the same hue (for instance in a whitish pink), although the hue of both is simply red. On the other hand, one can also say that the color most representative of redness is a highly chromatic red. In linguistic terms, talk of a focal color as the most representative color of a category (“the best cues of the category,” according to Rosch’s prototypical classification; Rosch, 1973; Rosch et al., 1976) makes reference to the color with which the word “red” fits best. In fact, focal color is the color in which one sees what one considers the best red, not a color which belongs to the red hue, which is reddest because it is less blue and less yellow. It is worth noting that the “best” red, differently form “unique” red, can bear cultural connotations as well.

Highly chromatic colors belonging to a bipolar scale between two consecutive hues show different degrees of similarity with the extreme colors of that interval. For instance, the interval defined by the extremes “most chromatic yellow” and “most chromatic red” in which mixed colors appear more or less yellowish or more or less reddish – i.e., are similar to one or the other color in different ways – show different degrees of similarity with the extreme colors of that interval. Linguistically, these intermediate colors can be expressed, for example, in terms of “red and yellow,” “saffron,” “pumpkin,” “orange,” “carrot,” etc. Not necessarily, however, do these color terms have the same referent, and some may also overlap. For example, a color may appear more or less red either because it is pink or because it is orange: in the former case, the hue is maximally red but little visible (the color is only slightly chromatic); in the latter case, the hue is not very red and the color may be highly chromatic. Consequently, one assesses pink as “very red” because it is only slightly or not at all yellow or blue; and likewise one assesses orange as only slightly red because the “hue” is not very red. However, it seems that one can also make an absolute assessment of how much a color is red, so that orange and pink might be treated equivalently, i.e., the extent to which red (not hue) is visible in them.

The perceptual similarity of the mixed hues to the extremes “red” and “yellow” can be quantified (for instance, halfway in the interval (50–50); or more yellowish than reddish (say, 70–30); and so on. Needless to say, different similarity metrics can be developed.

The problem of the perceptual identification and denomination of colors is particularly complex in the case of mixed colors, such as orange. To be noted is that Berlin and Kay’s (1977; see also Kay and Maffi, 1999) eleven basic color terms include both unique colors such as white, black, red, yellow, green, and blue (the first six colors in their list), and mixed colors such as orange. According to Sternheim and Boynton (1966), however, when the orange response category is available in a judgment experiment on the color continuum together with the response categories for red, yellow, and green, orange is used with the lowest reliability, i.e., randomly. When the orange response category is omitted, the hues otherwise associated with orange are completely dispersed into the red and the yellow, though with peaks in either red or yellow. Sternheim and Boynton (1966) therefore conclude that orange is some combination of red and yellow, and that the hues associated with the long wavelength part of the spectrum^3^ can be described without the category of orange, and making use of two already known color terms (yellow and red). The superfluous nature of the category “orange” was questioned by Boyton himself in a later study. He interviewed Japanese subjects, who were required to express their degree of agreement on the existence of specific categories related to Berlin and Kay’s basic color terms. For 90% of the subjects, the category of orange was well categorized as a salient color, and the category was linguistically expressed by mono-lexemic typical terms different from red and yellow (Uchikawa and Boynton, 1987).

This would imply that, phenomenologically, “orange” lies *exactly midway* between the two pure colors of red and yellow (on the status of “orange” from the point of view of painters, see Garau, 1993). Whenever orange varies from the mid-point between red and yellow, the resulting color is described as yellowish red or reddish yellow, as are the other mixed hues of the same range.

## NOMENCLATURES

One of the problems raised by the relationship between color perception and color terms is whether perceptual categorization requires linguistic categories at all. That is: do perceptual categories depend on language, learning and higher cognition, or are they independent from them? Munsell chips are definitely too poor a tool with which to verify this issue experimentally (Lucy and Shweder, 1979; Wierzbicka, 1996, 2006; Lucy, 1997). Testing the possible influence of language on color perception requires a more sophisticated experimental setting, such as having several words available for, say, red, in order to signal different environmental conditions (Green-Armytage, 2006; Winawer et al., 2007). In fact, as we have already noted, there is an indefinite number of color appearances, more than any natural language may encode. Therefore, the question arises as to how to relate natural language terms for perceived colors and the terminology adopted by scientific theories.

Scientific nomenclatures usually adopt severely constrained sets of basic terms and qualifiers. Four different spaces should be taken into account: (1) The space of colorimetry (to be noted, however, is that there are colorimetric spaces, such as CIELAB and CIECAM (respectively Lab Color Space and Color Appearance Model both published by CIE), that (do not perfectly) represent perceived colors, (2) the physiological space LMS (color space based on human cone cells – LMS stands for L- M- and S-cones) and its derivate DKL (Derrington–Krauskopf–Lennie color space), (3) the space of the linguistic representation of colors, and (4) the space of the subjective perception of colors.

To be noted is that the phenomenological perspective under (4), thus far rarely adopted, is starting to attract attention (Sivik, 1974, 1997; Albertazzi et al., 2012).

For each of these spaces, different theories are customarily developed. Each space requires specific groups of observables. The main issue is that most of the contemporary literature fails to distinguish them as clearly as needed, and therefore has difficulties in addressing the problem of their relations. Since colors, whatever they are, are also, and we would say primarily, a question of perception, one may wonder whether starting from real (i.e., subjective) perceptual experience of color provides information that may escape or remain hidden if one instead starts from other frameworks.

## COLOR PRIMITIVES

Color theories use different primitives – and even when they use the same terms, they may define them differently. It is consequently mandatory to be clear about the different terminologies and the ways in which different theories use any given term.

It is generally assumed that color can be described according to the parameters of hue, brightness and saturation (Kuehni, 2003; on measurement see Krantz et al., 1989)^4^. These properties make explicit reference to the relation between a given stimulus (hue correlated with wavelength, brightness correlated with luminance, saturation correlated with purity) and the subsequent subjective experience of a perceiver. On the other hand (see above), it is also often taken for granted that hue, brightness, and saturation are attributes of the color *as perceived*; also taken for granted is what they are correlated with, and what they correspond to; and that they form a 3D space where each of them represents a distinct dimension. These parameters result from innumerable experiments on the physical stimuli, i.e., light spectra, or the power at each wavelength. As it happens, light spectra can be readily measured and characterized by three numbers (the so-called tristimulus values of light). However, the shift is constantly made from properties of light spectra (as measured by the tristimulus values) to properties of the surfaces of seen objects (Wyszecki and Stiles, 1982; Hurlbert, 2013). It is customarily claimed that the tristimulus values specify the response of the standard human eye to the color spectrum. This standard response, however, is far from providing a *general* answer to the ways in which human eyes perceive colors, because the determination of the tristimulus values requires highly specific and severely constrained conditions, i.e., generally isolated colors. To provide an example, visual perception in complex environments where phenomena of contrast and assimilation regularly occur is purposely never taken into consideration: in fact, one of the major self-imposed limits adopted by colorimetric analysis is that it should consider only isolated colors, without taking colors combined with other colors into account (Boynton, 1979).

The problems are compounded because the literature on color defines hue, brightness, and saturation in different, often mutually incompatible, ways. Furthermore, although the distinction among hue, saturation and brightness is correct as far as the properties of light are concerned, it is far from being a “natural” – i.e., “phenomenological” – distinction from the point of view of the perceiver (Stumpf, 1917, p. 8; Katz, 1935). Saturation, for example, is a technical term used to characterize decontextualized light stimuli. According to the CIE definition of saturation, it is “the colourfulness of an area judged in proportion to its brightness” [1136], and in a note it is specified that “For given viewing conditions and at luminance levels within the range of photopic vision, a colour stimulus of a given chromaticity exhibits approximately constant saturation for all luminance levels, except when the brightness is very high.” Originally introduced by Helmholtz (1867) – explicitly aware of its arbitrariness from a perceptual point of view – the property of saturation should measure the degree of chromatic content in proportion to the brightness of a color. However, Wyszecki and Stiles (1982) note that the concept of saturation (together with the concept of chroma) is perhaps the most controversial concept in the literature on color appearance. In fact, different systems of color representation differ as to their primitives: for example, one finds chroma in Munsell and Sättigung in Deutsches Institut für Normung (DIN)^5^. Since the definition of saturation changes relatively to the color model adopted, the “‘usual” definition of saturation as the “colorfulness” (Hunt, 1986) of a color in relation to its “brightness,” or the degree of departure from the gray with the same lightness (all grays having zero saturation), is of little help (Mausfeld, 2003).

Finally, the different meanings of “saturation” or “chroma” are not limited to the different color systems in which they appear. Saturation, in fact, is confused with another phenomenological aspect of color, its insistence or forcefulness, i.e., the fact that a color appears more vivid or brighter in the field (Katz, 1935). These qualities of color carry emotional and affordance-type information like the difference between cold and warm colors (Ou et al., 2004a,b; Xin et al., 2004; Da Pos and Green-Armytage, 2007; Da Pos and Valenti, 2007) and the difference between light and heavy, large and small colors (Arnheim et al., 1954/1974; Itten, 1961), and they concern the theory of the harmonic dimensions of color (Burchett, 2007).

In past years, “brightness” was sometimes even used as synonymous with “lightness,” which fortunately is no longer the case. From a perceptual point of view, “brightness” is an attribute of the light that reaches the eye from a surface, while “lightness” refers to the colors of an object, i.e., it is an attribute of a surface. Lightness is an observable referring to white, understood as the color with the highest lightness (100%). It follows that the lightness of chromatic colors and grays is always less than 100%. Lightness then corresponds to the reflectance of a surface, a property of distal stimulus – that is, a phenomenologically inaccessible property. Using brightness and lightness as synonymous would therefore merge two different observables: an observable of light and an observable of surface. To further compound the confusion, “brightness” may be also used for surfaces, thereby indicating the more or less strong illumination (i.e., *light*) to which they are subject. In this latter case, many technicians prefer to use “luminance” (thereby not referring to the corresponding perception, i.e., brightness). Luminance, in fact, is a psychophysical property pertaining to the stimulus, and not perceivable as such by a perceiver.

Finally, “brightness” is also used for the correlation between the impression of lightness and luminance, where under the same luminance colors of higher saturation appear brighter than colors of low saturation (for example, the Helmholtz, Kohlrausch, and Boswell illusion; see Kaiser, 1985).

## FRAMEWORKS OF ANALYSIS

The foregoing discussion has shown how tangled the “scientific” analysis of colors is, and we have provided some evidence about how different some of the presently most widely used theories and approaches are. Some of their differences are due to pragmatic factors such as the needs of the communities using them: for instance, technicians requiring colorimetric data prefer to use either the DIN, the Munsell, the CIELAB or CIECAM02 systems (nowadays with a preference for the last). In one way or another, all the systems need to take account of four different natural systems: physical radiation, physiological elaboration, perception, and language. They differ as to where the focus falls, and therefore in which other system(s) should be kept under control in order to obtain the information they deem relevant. Munsell, NCS, and also OSA-UCS (Optical Society of America, Uniform Color Scale), for example, have a phenomenological base, none of them is primarily focused on physical radiation. Munsell, however, accepting the Fechnerian psychometric law adopts a two-sided understanding of perception, while the NCS adopts and develops a properly phenomenological stance (perception as connected to what appears to awareness), though ruled by psychometric principles.

The Munsell system constrains both psychological and linguistic information: the former by showing individual chips, that is by avoiding contextual influences on color, and the latter by admitting only yes/no answers by the perceiver.

On the other hand, the NCS constrains the neurophysiological base of perception and considers both the source and the neuronal elaboration of the stimuli to be irrelevant. This is not to imply that opponency has no neuronal correlates (Jameson and Hurvich, 1955; MacLeod and van der Twer, 2003; MacLeod, 2010). The problem, however, is that anatomo-physiological substrates cannot explain the phenomenological *qualities* of opponent colors (Valberg, 2001; Kuehni, 2004). As a matter of fact, stimuli for the NCS may arise from any source whatsoever (either “external” or “internal”), and there may be different kinds of them.

By not constraining its phenomenological base, NCS seems to better exploit the richness of both perceptual experience and its linguistic formulation: for example, the relation between warm and cold colors and its linguistic expression (Hård and Sivik, 1981; Da Pos and Valenti, 2007). The very existence of NCS shows that phenomenological observables can produce scientifically exploitable models of color.

The problem remains of making sense of the variety of models. As said, some models are explicitly tailored to the needs of specific communities of users, whilst others are more general in nature. The question however is that all the major models succeed in capturing aspects of the enormously complex problem of color perception. Finding a way to better codify the specific points of view embedded in the various models and systematically coordinate their outcomes may greatly deepen our understanding of colors. Since the discussion has already shown not only that the different models focus on different types of information, but that these types pertain to different sciences (physics, biology, psychology, linguistics – what in a more explicit philosophical parlance becomes the different levels of reality characterizing physical waves, neurophysiological activities, perception, and language – the question arises naturally whether a theory of the levels of reality will indeed be able to clarify and connect, at least to some degree, the different models.

## APPROACHING LEVELS OF REALITY

Before presenting some aspects of the theory of levels of reality and their relevance to our topic, some preliminary clarifications on the nature of ontological categories are needed. Needless to say, these clarifications are far from being anything like the presentation of a full-fledged, ontological framework. The economy of the paper forces us to skip issues that a purely philosophical paper would have to address. With these limitations, these clarifications may provide the required anchor points to an ontological framework sufficiently general to clarify scientific models.

We distinguish between categories on the one hand, and individuals on the other, as the entities to which categories refer. Only individuals pertain to the furniture of the world. Categories are not new entities added to the furniture of the world; they are instead principles (or determinations) of the individuals that they categorize. Individuals may be subdivided between *concreta* and *abstracta*, and their categories between real and ideal categories. *Concreta* and real categories pertain to the ontology of real being, *abstracta* and ideal categories to the ontology of ideal (or abstract) being. Universal categories comprise both *concreta* and *abstracta*, real and ideal being. The partial ontology that we are presenting in this paper deals with some aspects of real being only, namely colors.

Moreover, the difference between the nature of categories as principles and the often cumbersome process of their discovery and refinement should never be forgotten. The following quotation aptly summarizes our own understanding of ontological categories:

“the categories with which … ontology deals are won neither by a definition of the universal nor through derivation from a formal table of judgment. They are rather gleaned step by step from an observation of existing realities. And since, of course, this method of their discovery does not allow for an absolute criterion of truth, here no more than in any other field of knowledge, it must be added that the procedure of finding and rechecking is a laborious and cumbersome one. Under the limited conditions of human research it requires manifold detours, demands constant corrections, and, like all genuine scholarly work, never comes to an end”

(Hartmann, 1975, p. 13–14).

One of the most difficult problems faced by any ontology is the answer to the following question “What are the individuals to which ontological categories refer?” Two main positions compete; one according to which ontological individuals are only atomic entities, and one which accepts both atomic and molar entities. The former position sees ontological categories as referring to the most elementary components of the universe of discourse (e.g., colors as captured by colorimetry), from which all the other components should derive by composition or other suitable procedures. This is obviously the classic reductionist credo. The alternative vision is more flexible in the sense that it admits a variety of ontological individuals, some of which may work at molar levels of reality (e.g., colors as they appear in the environment, according to phenomena of assimilation and contrast). The main problem facing this alternative vision is that no generally accepted set of intermediate levels arise as the natural candidates from which to start. To compound the difficulty, the various sciences are such that a number of different levels present themselves as “natural” starting points. Selecting any one of them rather than any other is entirely arbitrary. Therefore, there is no saying that the former position is much simpler and (apparently) more effective than the latter. Notwithstanding all the difficulties encountered by the reductionist strategy, many see the reduction to atoms or basic individuals as a perhaps awkward but unavoidable TINA (There Is No Alternative) position. The underlying belief is that the difficulties arising from the reduction to atoms will eventually be solved by more refined strategies, such as new forms of composition. The possibility is usually overcome that even if some individual problem can be reductionistically analyzed, this does not necessarily imply that a generic (that is universal) reductionist strategy is available. Anyway, no patent decision procedure exists to help seriously puzzled scholars to choose between the former and the latter strategy. The unavailability of a proper decision procedure means that in the end the decision depends on a choice that the community of scholars has to take.

Our take on the issue is that the constraint forcing ontological categories to refer to atoms only impoverishes reality in the sense that information is lost and in the end authentic aspects of reality are missed. Instead, an ontological framework acknowledging both atomic and molar categories is both *more general*, in the sense of being able to categorize a wider spectrum of real phenomena, and *more complex*, in the sense of having to address many more problems, such as the ontological nature of the relations between different levels of reality.

This ontological framework systematically distinguishes between “pure” (i.e., “general” or “universal”) categories and “domain” (or “level”) categories. Keeping in mind this distinction will avert misunderstandings, especially when categories like those of space, time, and causation are introduced.

## LEVELS OF REALITY

Today, levels of reality are mostly discussed under the rubrics of “emergence” and “parts and wholes^6^.” In fact, the two most obvious strategies with which to approach levels are to divide the world into hierarchies of entities (such as atom–molecule–cell, etc.) or groups of properties (physical, biological, etc.). Not surprisingly, the main distinction among theories of levels of reality closely replicates the divide between entity-based and property-based theories. It is also not surprising that the entity-based theory of levels comes close to part-whole theories, and the property-based theory of levels comes close to type theories. Their merits and demerits notwithstanding, it is worth taking immediate note of an underlying problem: in the above lists of entities/properties, the exact meaning of the concluding “etc.” is unclear. Consider the entity-based framework: let us suppose that the series “atom–molecule–cell” will be at some point enlarged by the addition of new entities such as “mind” or “society” (or suitable alternatives). While there are *prima facie* plausible candidates for the relation connecting the items “atom,” “molecule,” and “cell” (e.g., a part–whole relation), the candidate relations for the new items are remarkably less easy to detect. Similarly, the connections between the properties characterizing “physical” and “biological” types are much simpler (e.g., a subset-set inclusion) than the connections between the properties characterizing the group comprising also “psychological” and “social” types^7^.

Of the two main ontological acceptations of entity-based or type-based theories of levels, the former, as said, comes close to the theory of parts and wholes, and the latter to the theory of ontological types. Let us adopt the latter option and understand a level of reality as a group of (ontological) categories (Poli, 2001).

The next step is to distinguish universal categories, those that pertain to the whole of reality, from level categories, those that pertain to one or more levels, but not to all of them. The distinction among physical, biological, psychological, and social types follows naturally. The subsequent step is to specify the relations connecting the levels to each other. Contemporary theories of levels of reality customarily exploit only *one* inter-level relation (e.g., in the form of supervenience). As far as color is concerned, for instance, its phenomenic appearance would be a supervenient product over its physical basis. One of the reasons for rehabilitating Hartmann’s theory of levels (see note 6) is that his theory uses *two* different inter-level relations and is therefore able to better distinguish the differences between the physical and the biological levels, on the one hand, and the biological and the psychological levels on the other (Poli, 2006a,b,c, 2007). Provided that the theory is fully developed and updated to contemporary knowledge, the two relations cover the connections between the physical and the biological levels, on the one hand, and among the biological, psychological, and social (including language and culture) levels on the other (Birren, 1969; Bornstein, 1973). With reference to colors, the two mentioned relations respectively cover stimuli (wavelengths) and their neuro-physiological elaboration (neural correlates), on the one hand, and perceptual modes of appearances of colors (Katz, 1935) and the relations among color terms in natural languages on the other.

As said, the original theory of levels developed by Hartmann is based on *two* different inter-level relations. Leaving universal categories aside, the following two main categorical situations can be distinguished: (a) Beings A and B are categorically different because the categories upon which the former is founded are partially different from the categories upon which the latter is founded, in the sense that the latter is founded on new categories (which implies that the latter includes at least a *novum*, a new category not present in the former); (b) Beings A and B are categorically different because the categories upon which the former is founded and those upon which the latter is founded form two entirely different (disjoint) groups of categories. Following Hartmann, the two relations can be termed respectively relations of super-formation (*Überformung*) and super-position (*Überbauung*; Hartmann, 1940).

Super-formation [the type (a) form of dependence] is weaker than super-position because it includes already actualized categories, those of the level below. Suffice it to consider the super-formation between molecules and cells, i.e., between the physical and the biological levels of reality. In this regard, one can mention that even if organisms are unquestionably more complex than mechanisms, the behavior of organisms complies with the laws of mechanics. On the other hand, the psychological and social levels are different because they are characterized by an interruption in the categorical series and by the onset of new categorical series (relative respectively to the psychological and social levels). The relation between the biological level and the psychological level, on the one hand, and the relation between the psychological level and the social one, on the other, are both relations of super-position. By way of example, the group of categories embedded in psychological entities is different from the group of categories embedded in biological entities. Similarly, the group of categories embedded in social entities is different from the group of categories embedded in biological entities.

When the connecting relation is a relation of super-formation, some categories of the lower level recur in the higher one. Recurring categories interact with the categories of the higher level and are, so to speak, contaminated by them; some of their moments become different. Higher levels are never characterized by recurring categories, however. Each level has its *novum*, the category or group of categories that distinguishes the level from the lower ones. The *novum* does not derive either from the elements of the level or from their synthesis.

Two aspects characterize super-position relations: first, the categories embedded in the entities of the connected levels are different (they are all *nova*); second, a relation of existential dependence links the higher level to the lower one. Most details of the links connecting together the various levels of reality are still unknown, because the various sciences have worked mainly on causal links internal to their regional phenomena^8^.

As an observable, color has ramifications into all these different levels of reality and as we have seen the properties of color are different in the different levels.

This is the main reason for at least some of the differences among the different color models. Specifically, the distinction between super-formation and super-position plays a major role. While two different levels related by a relation of super-formation may indeed present the same category, the internal determinants of this category are nevertheless partially different because the category pertains to two different categorical groups: that is to say, it interacts with two different groups of categories. One may say that the category seems to presents an intrinsic ambiguity. We say “seems” because the ambiguity is not embedded in intrinsic features of the category but depends entirely on the observer’s shift between different levels of reality (connected by a relation of super-formation). Reading a physical category (the three stimulus codification of a light wave) as a biological category (the three stimulus codification of a neural network) is a case in point.

On the other hand, levels of reality connected by a *super-position* relation present a remarkably different situation. In this latter case – and leaving universal categories aside – the categories defining the two levels are different. In this sense, no ambiguity is likely to arise. Moreover, the two levels are connected by a relation of existential dependence, meaning that the higher level requires the lower one as its existential bearer. Examples from the field of colors are provided by the difference between warm and cold, light and heavy, large and small colors (see Color Primitives above). None of these properties is present in the space of physical radiation. They are authentically phenomenological categories, present only at *that* level of reality. On the other hand, the phenomenological level requires suitable existential bearers – and more than one as a matter of fact: not only the brain as the bearer of the mind, but also the body (because the brain is not an autonomous whole)^9^, and the external environment. All of them are required, and all of them are sources of possible perceptual stimulation.

## CONCLUSION

As we have seen, color perception is paradigmatic for its complexity, including its ramifications into the physical, the neurophysiological, the linguistic (and cultural) and the phenomenological domains. Some of these ramifications are simpler than others. Not surprisingly, the phenomenological one is the most complex because phenomenic color exists only in the way in which it appears and therefore is a primarily contextual entity deeply influenced by interaction and assimilation (Katz, 1935) and language. The higher complexity of color perception may partly explain the preference shown by many experts for other points of view.

The research hypothesis that we have presented is that the theory of levels may clarify some of these intricacies in the sense of making explicit the ontological references of the various aspects of color, and it may therefore contribute to explaining the concepts of color used in science, phenomenology, and natural language conceptualization.

The analysis has shown that the different models explain color perception by encoding qualities pertaining to different levels of reality, which implies that strictly speaking they model different realities. However, since phenomenic color is essentially a contextual entity, the NCS system seems to be the model closer to color appearances. Further studies may provide additional evidence about whether the explicit connection between a model and the level of reality that it encodes is indeed able to clarify the relations among models themselves (an issue that may be called “ontology as a framework for clarifying science”).

## Conflict of Interest Statement

The authors declare that the research was conducted in the absence of any commercial or financial relationships that could be construed as a potential conflict of interest.

## Footnotes

1 That is, the hues presented in his *Notation* book, see Munsell (1905).

2 That is, universal color categories assumed to be present in most languages, and in a highly constrained order; (see Berlin and Kay, 1977; Kay and McDaniel, 1978; Kay and Regier, 2006, 2007).

3 The expression in Sternheim and Boynton’s paper is unfortunate, because the study refers to “perceived” colors.

4 Alternative names for “saturation” are “colorfulness,” “intensity,” and “purity.” Munsell uses instead a different primitive, namely “chroma”; “chromaticness” in NCS. See below for a brief reconstruction of their meaning.

5 DIN is based on a circle of 24 color-hues, a saturation scale, and a darkness scale as a special parameter for establishing the relative brightness of non-self-illuminating colors (i.e., colors that are illuminated by an external source). See http://www.colorsystem.com/?page_id=948&lang=en.

6 In the English-speaking world, both strands of analysis have been stimulated by influential papers by Hilary Putnam – notably Oppenheim and Putnam (1958) and Putnam (1961). Since them, an enormous discussion has developed, which cannot be summarized here (for an old but still valuable survey, see Blitz, 1992). However, as important as the discussion in English has been, it is worth noting that some major pre-WWII contributions have never been taken into account, notably those by Nicolai Hartmann. See Hartmann (1940, 1975), Werkmeister (1990), Poli (2012).

7 Furthermore, beyond or above the distinction between entity-based and type-based theories of levels of reality, other acceptations of levels often intrude, such as notions of levels of organization, complexity or representation.

8 The lack of a theory of levels of reality has possibly been the main obstruction against development of the theories needed. Proposals concerning the architecture of levels and their links will improve our understanding of the world and its many dependencies. To mention but one case, the theory of levels paves the way to the claim that there may be different families of times and spaces, each with its own structure. We shall argue that there are numerous types of real times and spaces endowed with structures that may differ greatly from each other. The qualifier *real* is mandatory, since the problem is not the trivial one that different abstract theories of space and time can eventually be, and have been, constructed. We shall treat the general problem of space and time as a problem of chronotopoids (understood jointly, or separated into chronoids and topoids). The guiding intuition is that each stratum of reality comes equipped with its own family of chronotopoids (Poli, 2007; for further details on the theory of levels of reality, see Poli, 1998, 2001, 2006a,b,c, 2009, 2010a,b, 2011a,b, 2012).

9 Here is where the connection with the theory of levels from the perspective point of the theory of wholes becomes visible.
